# Chloride of Soda for Burns, Ulcers, Etc.

**Published:** 1871-01

**Authors:** 


Chloride of Soda for Burns, Ulcers, &c.—The sudden relief of the pain of burns, which attends the application of a solution of half an ounce of the French Chloride of Soda in a quart of water, is truly astonishing. It should be applied by means of cloth dipped into it and kept wet. If it fails to give relief, you may suspect the impurity of the Soda, or that the solution is too strong. If its anti-phlogistic effect be desired, it must give no pain when applied. In cases tending to mortification, it may be best to apply it sufficiently strong to produce slight smarting. When too strong, nothing is more irritating and consequently injurious to a burn, new wound or ulcer; but when the French Chloride is used—half an ounce to the quart of water—we know of nothing better as an application or wash to recent wounds of a lacerated character, or to chronic ulcers. We would recommend it especially in children to prevent the excessive suppuration which is itself so often fatal in such cases.

